# Triple-negative breast cancer molecular subtyping and treatment progress

**DOI:** 10.1186/s13058-020-01296-5

**Published:** 2020-06-09

**Authors:** Li Yin, Jiang-Jie Duan, Xiu-Wu Bian, Shi-cang Yu

**Affiliations:** 1 Department of Stem Cell and Regenerative Medicine, Southwest Hospital, Third Military Medical University (Army Medical University), ChongQing, 400038 China; 2 Institute of Pathology and Southwest Cancer Center, Southwest Hospital, Third Military Medical University (Army Medical University), ChongQing, 400038 China; 3 Key Laboratory of Cancer Immunopathology, Ministry of Education, ChongQing, 400038 China

**Keywords:** Triple-negative breast cancer, Molecular subtype, Therapeutic target, Therapeutic regimen

## Abstract

Triple-negative breast cancer (TNBC), a specific subtype of breast cancer that does not express estrogen receptor (ER), progesterone receptor (PR), or human epidermal growth factor receptor 2 (HER-2), has clinical features that include high invasiveness, high metastatic potential, proneness to relapse, and poor prognosis. Because TNBC tumors lack ER, PR, and HER2 expression, they are not sensitive to endocrine therapy or HER2 treatment, and standardized TNBC treatment regimens are still lacking. Therefore, development of new TNBC treatment strategies has become an urgent clinical need. By summarizing existing treatment regimens, therapeutic drugs, and their efficacy for different TNBC subtypes and reviewing some new preclinical studies and targeted treatment regimens for TNBC, this paper aims to provide new ideas for TNBC treatment.

## Introduction

Breast cancer is the most common malignancy in women. The 2012 global cancer statistics showed that there were approximately 1.7 million women diagnosed with breast cancer, and 521,900 women died of breast cancer [1]. Breast cancer is a highly heterogeneous disease. Clinical treatment and prognosis varies greatly between patients. The 2013 St. Gallen International Breast Cancer Conference issued a new definition of breast cancer molecular subtypes: luminal A (ER/PR^+^, HER2^−^, Ki67^+^ < 20%, with the percentage indicating the immunohistochemical staining results for patient samples), luminal B (ER/PR^+^ < 20%, HER2^−^, Ki67^+^ ≥ 20%); HER2^+^ B2 (ER/PR^+^, HER2 overexpression), HER2 overexpression (ER^−^, PR^−^, HER2 overexpression), basal-like TNBC (ER^−^, PR^−^, HER2^−^), and other special subtypes [2].

Triple-negative breast cancer (TNBC) is defined as a type of breast cancer with negative expression of estrogen (ER), progesterone (PR), and human epidermal growth factor receptor-2 (HER2) [3]. Gene expression profiling analysis often classifies TNBC as a subtype of basal-like breast cancer (BLBC). Approximately 56% of TNBC and BLBC gene expression profiles overlap. The overlap ratio can be as high as 60–90% between TNBC and BLBC, compared to only 11.5% between non-TNBC and BLBC [4, 5].

Epidemiological data show that TNBC mostly occurs in premenopausal young women under 40 years old, who account for approximately 15–20% of all breast cancer patients [6]. Compared with other subtypes of breast cancer, the survival time of TNBC patients is shorter, and the mortality rate is 40% within the first 5 years after diagnosis [7]. TNBC is highly invasive, and approximately 46% of TNBC patients will have distant metastasis. The median survival time after metastasis is only 13.3 months, and the recurrence rate after surgery is as high as 25%. The metastasis often involves the brain and visceral organs. Distant metastasis mostly occurs in the 3rd year after diagnosis [8]. The average time to relapse in non-TNBC patients is 35–67 months, while that in TNBC patients is only 19–40 months. The mortality rate of TNBC patients within 3 months after recurrence is as high as 75% [9, 10].

Due to its special molecular phenotype, TNBC is not sensitive to endocrine therapy or molecular targeted therapy. Therefore, chemotherapy is the main systemic treatment, but the efficacy of conventional postoperative adjuvant chemoradiotherapy is poor. The residual metastatic lesions eventually will lead to tumor recurrence [11]. Bevacizumab has been used in combination with chemotherapeutic drugs to treat TNBC in some countries, but the survival time of patients did not increase significantly [12]. Therefore, it is urgent to develop new treatment regimens and targets.

This paper reviews existing treatment regimens, therapeutic drugs, and their efficacy in treating TNBC patients and discusses some new preclinical studies and targeted treatment regimens, aiming to provide new ideas and directions for TNBC treatment regimens and targets.

## TNBC subtyping and treatment regimens

In 2011, Lehmann et al. performed gene expression profiling of tumor samples from 587 TNBC patients and divided TNBC into six subtypes: basal-like 1 (BL1), basal-like 2 (BL2), mesenchymal (M), mesenchymal stem-like (MSL), immunomodulatory (IM), and luminal androgen receptor (LAR) [13]. They also performed gene profiling and compared existing TNBC breast cancer cell lines, classifying them into six different subtypes, thus providing an accurate cell model for clinical treatment of TNBC (Table 1).

**Table 1:** Genomic TNBC subtypes and assignment of TNBC cell lines to subtypes

TNBC subtype	Genetic abnormalities	Cell line	Subtype correlation^A^ (*p* value)	Histology	Mutations
Basal-like 1	Cell cycle gene expression DNA repair gene (ATR-BRCA pathway) Proliferation genes	HCC2157 HCC1599 HCC1937 HCC1143 HCC3153 MDA-MB-468 HCC38	0.66 (0.00) 0.62 (0.00) 0.28 (0.00) 0.26 (0.00) 0.24 (0.00) 0.19 (0.06) 0.19 (0.01)	DC DC DC IDC DC DC	BRCA1; STAT4; UTX BRCA2; TP53; CTNND1; TOP2B; CAMK1G BRCA1; TP53; MAPK13; MDC1 TP53 BRCA1 PTEN; RB1; SMAD4; TP53 CDKN2A; TP53
Basal-like 2	Growth factor-signaling pathways (EGFR, MET, NGF, Wnt/β-catenin, IGF-1R) Glycolysis, gluconeogenesis Expression of myoepithelial markers	SUM149PT CAL-851 HCC70 HCC1806 HDQ-P1	0.30 (0.00) 0.25 (0.00) 0.24 (0.04) 0.22 (0.00) 0.18 (0.17)	INF IGA DC ASCC IDC	BRCA1 RB1; TP53 PTEN; TP53 CDKN2A; TP53; UTX TP53
Immunomodulatory	Immune cell processes (CTLA4, IL2, IL7 pathways, antigen processing/presentation) Gene signature for medullary BC (rare TNBC with a favorable prognosis)	HCC1187 DU4475	0.22 (0.00) 0.17 (0.00)	DC DC	TP53; CTNNA1; DDX18; HUWE1; NFKBIA APC; BRAF; MAP 2 K4; RB1
Mesenchymal-like	Cell motility Cell differentiation Growth factor signaling EMT	BT-549 CAL-51 CAL-120	0.21 (0.00) 0.17 (0.00) 0.09 (0.00)	IDC AC AC	PTEN; RB1; TP53 PIK3CA TP53
Mesenchymal stem-like	Similar to M+ Low proliferation Angiogenesis genes	HS578T MDA-MB-157 SUM159PT MDA-MB-436 MDA-MB-231	0.29 (0.00) 0.25 (0.00) 0.14 (0.00) 0.13 (0.00) 0.12 (0.00)	CS MBC ANC IDC IDC	CDKN2A; HRAS; TP53 NF1; TP53 PIK3CA; TP53 HRAS BRCA1; TP53 BRAF; CDKN2A; KRAS; NF2; TP53; PDGFRA
Luminal androgen receptor	Androgen receptor gene Luminal gene expression pattern Molecular apocrine subtype	MDA-MB-453 SUM185PE HCC2185 CAL-148 MFM-223	0.53 (0.00) 0.39 (0.00) 0.34 (0.00) 0.32(0.00) 0.31 (0.00)	AC DC AC AC	PIK3CA; CDH1; PTEN PIK3CA PIK3CA PIK3CA; RB1; TP53; PTEN PIK3CA; TP53

Data from Lehmann et al. [14]

*Abbreviations*: *AC* adenocarcinoma, *ANC* anaplastic carcinoma, *ASCC* acantholytic squamous cell carcinoma, *CS* carcinosarcoma, *DC* ductal carcinoma, *IDC* invasive ductal carcinoma, *IGA* invasive galactophoric adenocarcinoma, *INF* inflammatory ductal carcinoma, *MC* metaplastic carcinoma and *MBC* medullary breast cancer

^A^Gene expression (GE) data for TNBC cell lines (GSE-10890 and E-TABM-157) were correlated (Spearman) to the centroids of the GE signatures for each TNBC subtype. GE data from both the TNBC tumors and cell lines were combined so that each gene was standardized to have mean = 0 and SD = 1. GE profiles from the cell lines were correlated to the centroids for each of the 6 TNBC subtypes. Cell lines were assigned to the TNBC subtype with the highest correlation, and those that had low correlations (< 0.1) or were similar between multiple subtypes (*p* > 0.05) were considered unclassified

Gene expression profiling analysis of TNBC tumor samples found abnormal expression of cell cycle-regulating and DNA repair-related genes in the BL1 subtype (high amplification of *MYC*, *PIK3CA*, *CDK6*, *AKT2*, *KRAS*, *FGFR1*, *IGF1R*, *CCNE1*, and *CDKN2A*/B and high frequency of heterozygous or homozygous deletion of DNA repair-related genes such as *BRCA2*, *PTEN*, *MDM2*, *RB1*, and *TP53*). Possible therapeutic drugs for the BL1 subtype include poly (ADP-ribose) polymerase (PARP) inhibitors and genotoxic agents. BL1 patients were sensitive to cisplatin treatment [14]. The BL2 subtype has abnormal activation of signaling pathways such as the EGFR, MET, NGF, Wnt/β-catenin, and IGF-1R pathways, and the potential targeted therapeutic drugs include mTOR inhibitors and growth factor inhibitors (lapatinib, gefitinib, and cetuximab) [14].

The M subtype has highly activated cell migration-related signaling pathways (regulated by actin), extracellular matrix–receptor interaction pathways, and differentiation pathways (Wnt pathway, anaplastic lymphoma kinase pathway, transforming growth factor (TGF)-β signaling) and is therefore also called metaplastic breast cancer [14]. The M subtype has sarcoma-like or squamous epithelial cell-like tissue characteristics and is prone to develop resistance to chemotherapeutic drugs. Therefore, M-subtype patients might be treated with mTOR inhibitors or drugs targeting epithelial–mesenchymal transition [15].

Compared with the M subtype, the MSL subtype expresses low levels of cell proliferation-related genes and high levels of stemness-related genes (*ABCA8*, *PROCR*, *ENG*, *ALDHA1*, *PER1*, *ABCB1*, *TERT2IP*, *BCL2*, *BMP2*, and *THY*), HOX genes (*HOXA5*, *HOXA10*, *MEIS1*, *MEIS2*, *MEOX1*, *MEOX2*, and *MSX1*), and mesenchymal stem cell-specific markers (*BMP2*, *ENG*, *ITGAV*, *KDR*, *NGFR*, *NT5E*, *PDGFR*, *THY1*, and *VCAM1*). It is speculated that the MSL subtype patient may be treated with PI3K inhibitors, Src antagonists, or antiangiogenic drugs. Studies have reported that the dasatinib, an Abl/Src inhibitor, can be used for the treatment of patients with M and MSL breast cancers [14].

The IM subtype has significantly enriched immune cell-associated genes and signal transduction pathways, such as the Th1/Th2 pathway, NK cell pathway, B cell receptor signaling pathway, dendritic cell (DC) pathway, T cell receptor signaling, interleukin (IL)-12 pathway, and IL-7 pathway. Thus, the IM subtype is highly similar to medullary carcinoma of the breast [16]. It is recommended to use PD1, PDL1, CTLA-4, and other immune checkpoint inhibitors for the treatment of patients with IM subtype breast cancer [14].

The LAR subtype has a significantly different gene expression profile than other TNBC subtypes. Although the LAR subtype does not express ER receptor, it does have highly activated hormonal-related signaling pathways (including steroid synthesis, porphyrin metabolism, and androgen/estrogen metabolism). It is worth noting that androgen receptor (AR) is highly expressed in the LAR subtype of breast cancer, and its mRNA level is nine times that in other TNBC subtypes. Immunohistochemistry also detects high expression of AR and a large number of downstream metabolic markers of AR and their auxiliary activators (DHCR24, ALCAM, FASN, FKBP5, APOD, PIP, SPDEF, and CLDN8) in the LAR subtype [17]. Therefore, anti-AR therapy is recommended for patients with LAR-subtype breast cancer.

Lehmann et al. performed PAM50 subtyping of the six TNBC subtypes and compared their PAM50 molecular intrinsic subtypes. It was found that, other than the LAR and MSL subtypes, all TNBC subtypes were mainly composed of the basal-like subtypes (BL1 [99%], BL2 [95%], IM [84%], and M [97%]). LAR subtypes included HER2 (74%) and luminal B (14%); MSL subtypes included basal-like (50%), normal-like (28%), and luminal B (14%) [14]. Masuda et al. performed a prognostic analysis of different TNBC subtypes and found that the LAR subtype had a higher distant metastasis-free survival rate and overall survival rate (OS), while those of the M and BL2 subtypes were poorer. The 3-year recurrence rates of the M and BL2 subtypes were significantly higher than that of the LAR subtype [18]. In addition, retrospective analysis of 130 TNBC patients who received anthracycline and paclitaxel chemotherapy showed that although the overall pathologic complete remission (pCR) rate was 28%, there were significant differences in the specific responses between subtypes. The pCR rate of the BL1 subtype was the highest (52%), while those of the BL2, LAR, and MSL subtypes were 0%, 10%, and 23%, respectively [18].

Complementing the above molecular subtyping, Burstein et al. analyzed samples from 198 patients and divided TNBC into four subtypes: LAR, expresses AR and cell-surface mucin MUC1; M, expresses growth factor receptors (platelet-derived growth factor receptor α [PDGFRα] and c-Kit receptor); BLIS (basal-like immunosuppressed), expresses the immunosuppressive molecule VTCN1; and BLIA (basal-like immune-activated), expresses STAT signal transduction molecules and releasing cytokines. The prognosis analysis showed that disease-free survival (DFS) was in the order BLIA > M > LAR > BLIS (*p* = 0.019) and disease-specific survival (DSS) was BLIA > M > LAR > BLIS (*p* = 0.07) [19].

What has not yet been determined is whether the TNBC molecular subtypes are associated with disparities in clinical outcome across race and ethnicity. To date, compared with Caucasian women, few non-Caucasian women have been included in studies defining TNBC subtypes, even though both Hispanic and African-American women account for a higher proportion of TNBC patients than Caucasian women [20]. Even within The Cancer Genome Atlas (TCGA) breast cancer set of more than 1000 women, few samples are from non-Caucasian women with TNBC. Using nCounter Gene Expression CodeSets, Ding et al. [21] classified TNBC into subtypes: BLIA, BLIS, LAR, and M in 48 Hispanic, 12 African-American, 21 Asian, and 34 Caucasian patients. No association was found between family history or race and ethnicity and the overall distribution of the four subtypes. In multivariate Cox proportional hazards modeling, Hispanic women made up a significantly higher proportion of BLIS patients (53%, *p* = 0.03), whereas Asian women made up a lower proportion of BLIS patients (19%, *p* = 0.05) and a higher proportion of LAR patients (38%, *p* = 0.06) compared with the average proportion across all groups. The Asian women in the study represented a significantly lower number of BLIS patients and a higher number of LAR patients and showed the best OS, which is consistent with what has been previously reported for survival [22].

Considering the emerging important role of long noncoding RNAs (lncRNAs) in cellular processes, a novel classification integrating both messenger RNA (mRNA) and lncRNA transcriptome profiles would help provide a better understanding of the heterogeneity of TNBC. After categorization analysis of 165 TNBC samples combined with mRNA expression analysis and coexpression network analysis to identify interactions between mRNAs and lncRNAs, Liu et al. [23] proposed a new definition of TNBC subtypes (Table 2). The immunomodulatory subtype (IM) expresses a unique Gene Ontology (GO) category profile and participates in the regulation of immune cells. These regulatory factors include cytokine signaling (the interaction between cytokines and cytokine receptors), immune cell signal transduction (the T cell receptor signaling pathway and the B cell receptor signaling pathway), antigen processing and presentation, chemokine signaling pathways, and immune signal transduction pathway (NF-κB signaling pathway). The genes highly expressed in the IM subtype are all associated with immune functions such as immune response, T cell costimulation, and innate immune response. Some genes that are involved in the immune response process (*CCR2*, *CXCL13*, *CXCL11*, *CD1C*, *CXCL10*, and *CCL5*) are also highly expressed, suggesting that the IM subtype TNBC is closely related to immune regulation. The LAR subtype has unique hormonal regulation pathways active, including androgen and estrogen metabolism, steroid hormones biosynthesis, and porphyrin and chlorophyll metabolism. The peroxisome proliferator-activated receptor (PPAR) signaling pathway is also significantly increased in the LAR subtype [13]. Although immunohistochemical analysis indicates that the LAR subtype is ER-negative, the signaling pathway expression profile shows the activation of the estrogen signaling pathway, suggesting that the LAR subtype may respond to antiandrogen and traditional antiestrogen therapy [19]. The mesenchymal-like subtype (ME) is characterized by a variety of unique GO category members and signaling pathways, such as extracellular matrix–receptor interactions, gap junctions, TGF-β signal transduction pathways, and pathways associated with growth factors (ABC transporters and adipokine signaling pathways). BLIS subtype: The GO terms enriched in the BLIS subtype are cell division, cell cycle, DNA replication, and DNA repair regulation. In the BLIS subtype, the expression of proliferation-related genes is significantly enhanced, including *CENPF*, *BUB1*, and *PRC1*. Thus, the BLIS subtype exhibits highly proliferative properties [19]. Genes involved in immune response (immune response and innate immune response), immune cell signaling (T cell costimulation, T cell receptor signaling pathway, B cell activation, and DC cell chemotaxis), and the complement activation process are significantly downregulated in the BLIS subtype. A prognostic analysis showed that patients with the BLIS subtype had a worse RFS and a higher risk of recurrence than other subtypes. These results are consistent with previous statistical results by Burstein et al. [23].

**Table 2:** TNBC subtypes based on the FUSCC classification criteria

FUSCC classification	GO terms/canonical pathway	Most upregulated lncRNAs	Correlated mRNA	TNBC cell line
IM	Cytokine-cytokine receptor interaction↑ T cell receptor signaling pathway ↑ B cell receptor signaling pathway ↑ Chemokine signaling pathway ↑ NF-kappa B signaling pathway ↑	ENST00000443397	LOC100653210, LOC100653245, IGHV3-20, IGHV4-31, IGHJ1, IGKV3-7.	MDA-MB-231
LAR	Steroid hormone biosynthesis ↑ Porphyrin and chlorophyll metabolism ↑ PPAR signaling pathway ↑ Androgen and estrogen metabolism ↑	ENST00000447908	TRIM2, SDR16C5, C1QTNF3, KRT17, SERPINB5, TFAP2B, FAR2, CYP39A1, KIAA1467, EDDM3B.	HS578T
MES	ECM-receptor interaction ↑ Focal adhesion ↑ TGF-beta signaling pathway ↑ ABC transporter ↑ Adipocytokine signaling pathway ↑	NR_003221	SELP, CNN1, ADH1B.	HCC1937
BLIS	Mitotic cell cycle↑ Mitotic prometaphase↑ M phase of mitotic cell cycle↑ DNA replication↑ DNA repair↑ Immune response↓ Innate immune response ↓ T cell receptor signaling ↓	TCONS_00000027	RNASE6, MS4A6A, MTBP, FGFR2, CXor161, DHTKD1, IGLV6-57, BARD1, PRTFDC1.	MDA-MB-436

Data from Yi-Rong Liu et al. [23]

*Abbreviations*: *FUSCC* Fudan University Shanghai Cancer Center, *IM* immunomodulatory, *LAR* luminal androgen receptor, *MES* mesenchymal-like, *BLIS* basal-like and immune suppressed, *BL* basal-like, *M* claudin-low-enriched mesenchymal, *MSL* mesenchymal stem-like, *ECM* extracellular matrix, *TGF* transforming growth factor

**Fig. 1:**
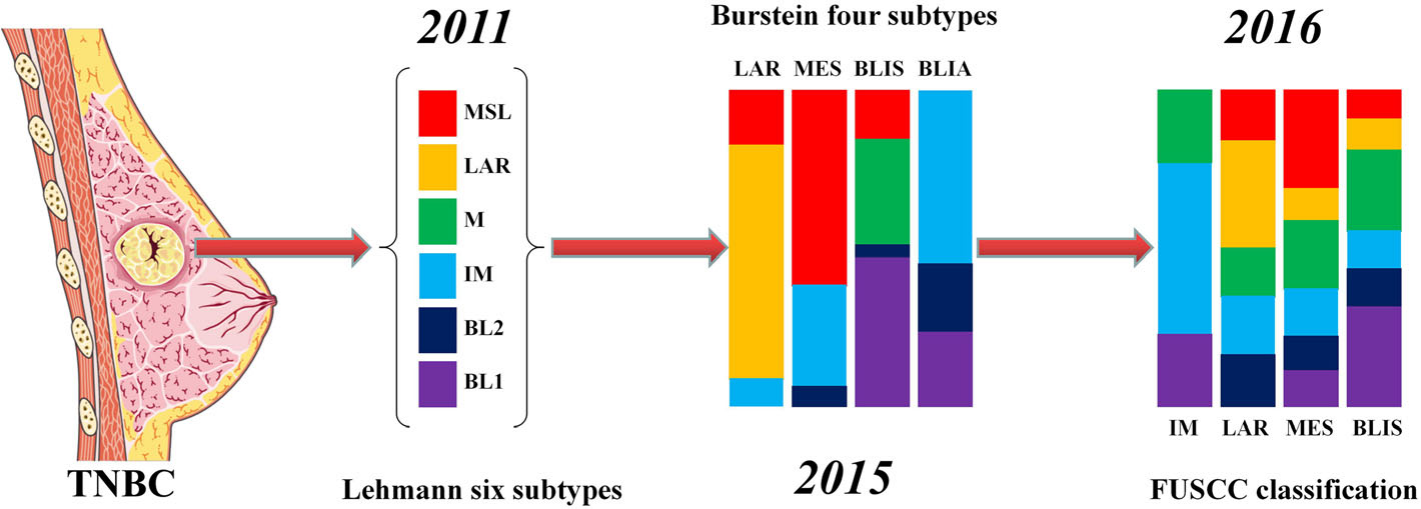
Progress in classification of TNBC molecular types, and interaction analysis of the Burstein four subtypes/FUSCC classification and Lehmann six subtypes, rectangle size varies in proportion to the number of samples [14, 19, 23]. AC, adenocarcinoma; ANC, anaplastic carcinoma; ASCC, acantholytic squamous cell carcinoma; CS, carcinosarcoma; DC, ductal carcinoma; IDC, invasive ductal carcinoma; IGA, invasive galactophoric adenocarcinoma; INF, inflammatory ductal carcinoma; MC, metaplastic carcinoma and MBC, medullary breast cancer

Through cluster analysis of different gene expression levels in a large number of samples of TNBC patients, researchers carried out accurate molecular subtyping of highly heterogeneous TNBC (Fig. 1). Currently, most of the studies on TNBC molecular subtyping are based on the mRNA levels of different genes. However, the mRNA expression level cannot accurately reflect the protein expression level, and there are many modification and regulatory steps in the protein translation process, which affect the targeted therapeutic effect and prognostic prediction in some patients. At the same time, how to accurately determine TNBC molecular subtype based on immunohistochemical staining results in the clinic and in terms of the TNBC clinical specimen numbers is still unclear, and the results are far from adequate. Therefore, different biomarkers associated with TNBC molecular subtype and their clinical definitions await further study. Perhaps, in future clinical practice, gene chip technology can be used to quickly determine the breast cancer molecular subtype in patients, and further, molecular analysis of protein expression in TNBC patient clinical specimens can be conducted to accurately reflect the TNBC phenotype and guide screening of targeted drugs.

## TNBC chemotherapy drugs and efficacy evaluation

Compared to other types of breast cancer, TNBC has limited treatment options, is prone to recurrence and metastasis, and has a poor prognosis. The main reason is that the expression of ER, PR, and HER2 are all negative, making specific endocrine therapies and targeted therapies ineffective. Therefore, chemotherapy has become the main approach for the treatment of TNBC. In recent years, a large body of literature has shown that the use of neoadjuvant chemotherapy regimens in the treatment of TNBC has a significantly higher pathological remission rate than for hormone receptor-positive breast cancer and can significantly improve the prognosis of TNBC patients. The national comprehensive cancer network guidelines recommend using combination regimens based on taxane, anthracycline, cyclophosphamide, cisplatin, and fluorouracil. At present, taxel/docetaxel + adriamycin + cyclophosphamide (TAC), docetaxel + cyclophosphamide (TC), adriamycin + cyclophosphamide (AC), cyclophosphamide + methotrexate + fluorouracil (CMF), cyclophosphamide + adriamycin + fluorouracil (CAF), and cyclophosphamide + epirubicin + fluorouracil + paclitaxel/docetaxel (CEF-T) are the preferred adjuvant regimens for TNBC. Therefore, the selection of appropriate chemotherapy drugs and the optimization of chemotherapy regimens are important for ensuring good treatment outcome and prognosis of TNBC patients.

### Taxanes

The mechanism of action of taxel is mainly through the inhibition of microtubule depolymerization, and thus, cells cannot form spindles and spindle fibers during mitosis, forcing the cells to stop in prometaphase, thereby inhibiting cell division. In addition to its antimitotic effect, taxel also has the antitumor function mediated by activated macrophages. The antitumor toxicity of taxel is also associated with its induction of apoptosis. The mechanism underlying the action of docetaxel is the same as that of taxel, but at the same toxic dose, docetaxel has twice the anti–microtubule depolymerization effect of taxel and has a broader antitumor spectrum. Further in-depth studies in recent years have found that conventional, commercially available solvent-based (Sb) taxel prepared using polyoxyethylated castor oil (Kolliphor® EL, formerly known as Cremophor EL; BASF SE, Ludwigshafen, Germany) as the solvent can cause severe or even fatal allergic reactions. The commonly used solvent polyoxyethylated castor oil severely restricts the release of taxel particles and reduces its efficacy [24]. Compared with sb-paclitaxel, albumin-bound paclitaxel (Nab-paclitaxel) can shorten the time of drug administration, does not need pretreatment to prevent allergic reactions, and has a higher drug delivery efficiency on endothelial cells [25]. Gene profiling analysis of TNBC molecular subtypes showed that the BL subtype has active expression of proliferation-related genes and DNA repair genes, suggesting that the BL subtype may be sensitive to antimitotic drugs (e.g., taxel or docetaxel). After the application of taxane-based chemotherapy in TNBC patients, the basal-like subtypes (BL1 and BL2) have four times higher clinical remission rates than the MSL subtype and LAR subtype [26, 27].

### Anthracyclines

Anthracyclines and anthracycline antibiotics are a class of chemotherapeutic drugs derived from *Streptomyces peucetius* var. *caesius*. They can treat more types of cancer than any other type of chemotherapeutic drug and can be used to treat leukemia, lymphoma, breast cancer, uterine cancer, ovarian cancer, and lung cancer [28]. Through a large number of clinical studies, researchers have obtained optimal dosing schedules of anthracycline adjuvant therapy for breast cancer: the optimum dose of doxorubicin is 60 mg/m^2^ and that of epirubicin is 100 mg/m^2^ [29]. Further studies showed that increasing the dose did not improve the survival rate or reduce the relapse rate [30]. Existing anthracycline-based regimens, such as FEC-100 (100 mg/m^2^ epirubicin), can reduce the risk of relapse and death from breast cancer by 25–30% [31, 32]. According to the existing clinical data, after 6 months of chemotherapy with anthracyclines, the mortality rate decreased by approximately 38% in patients younger than 50 years at the time of diagnosis, whereas the mortality rate in patients aged 50 to 69 years at the time of diagnosis decreased by approximately 20%. The efficacy of anthracycline chemotherapy showed no significant difference between breast cancer subtypes [33]. However, the specific responses to the combination of anthracyclines and taxanes vary greatly between subtypes. TNBC patients with the BL1 or MSL subtype have a higher rate of pCR, while the TNBC patients with the LAR and BL2 subtypes are not sensitive to the combination regimen. The pCR rate of patients with the BL2 subtype was 0%.

### Cyclophosphamide

Cyclophosphamide does not have antitumor activity in vitro. After entering the body, cyclophosphamide is first converted to aldophosphamide by the microsomal mixed-function oxidases in the liver. Aldophosphamide is unstable and is activated by cytochrome P450 in tumor cells to produce nitrogen mustard and acrolein with alkylating activity. Nitrogen mustard has cytotoxic effects on tumor cells. Currently, TC is commonly used as the standard neoadjuvant chemotherapy regimen for HER2-negative breast cancer. Nakatsukasa et al. enrolled 52 breast cancer patients. Of them, 94.2% (49/52) patients completed 4 TC cycles and had an overall pCR rate of 16.3% (8/49); patients with luminal A-like breast cancer (ER^+^, Ki67 index < 20%, HER2 negative) had a pCR rate of 0% (0/12); patients with luminal B-like breast cancer (ER^+^, Ki67 index > 20%, HER2 negative) had a pCR rate of 4.3% (1/23); patients with TNBC had a pCR rate of 50.0% (7/14); almost all of the pCR occurred in TNBC breast cancer patients [34]. The results showed that neoadjuvant chemotherapy with TC was more suitable for the treatment of TNBC but had limited efficacy in treating other subtypes of breast cancer. Wu et al. found that adjuvant cyclophosphamide, methotrexate, and fluorouracil chemotherapy effectively reduced the locoregional recurrence rate and prolonged the DFS of patients with node-negative TNBC, especially in patients with a tumor diameter greater than 2 cm and in patients who had undergone partial mastectomy [35]. Masuda et al. [18] previously performed a retrospective analysis of response rates by TNBC subtype in 130 TNBC cases treated with neoadjuvant adriamycin/Cytoxan/Taxol-containing chemotherapy. The overall pCR response was 28%, and interestingly, the specific responses differed substantially between subtypes. The BL1 subtype achieved the highest pCR rate (52%), and the BL2, LAR, and MSL subtypes were found to have the lowest response rates (0%, 10%, and 23%, respectively). TNBC subtype was also shown to be an independent predictor of pCR status (*p* = 0.022) by a likelihood ratio test [18]. These results speak not only to the heterogeneity of TNBC but also to the need to align patients to different therapies based on the subtype of their disease.

### Platinum agents

The cis-structured platinum compound, cisplatin, has inhibition effect on cancer cells [36]. Zhang et al. conducted a phase II study (NCT00601159) to evaluate the efficacy and tolerability of cisplatin and gemcitabine (GP) as the first-line treatment regimen of metastatic TNBC (mTNBC). The results showed that the combination regimen had significant activity and favorable safety for mTNBC patients, particularly patients with basal-like subtypes [37]. Von Minckwitz administered a carboplatin-containing treatment to 269 randomly selected breast cancer patients and a non-carboplatin-containing treatment to 299 breast cancer patients. They found that the addition of carboplatin to conventional taxel chemotherapy and anthracycline chemotherapy significantly increased the pCR rate in TNBC patients, but that increase was not observed in patients with HER2-positive breast cancer [38]. Other results showed that BL1-subtype TNBC had significantly higher sensitivity to cisplatin chemotherapy than other TNBC subtypes [39].

### Fluorouracil

5-Fluorouracil (5-Fu) itself does not have any biological activities. Under the action of orotate phosphoribosyltransferase, 5-Fu can be converted into active metabolites, fluorouridine monophosphate and fluorodeoxyuridine monophosphate, in vivo. Capecitabine is a cytotoxic agent that has selective activity against tumor cells. Capecitabine itself has no cytotoxicity and is highly effective after transforming into cytotoxic 5-Fu in vivo. This process is catalyzed by the large amount of thymidylate phosphorylase in the tumor, resulting in the production of more 5-Fu in the tumor, with stronger (better than 5-Fu) antitumor efficacy. Capecitabine is suitable for the further treatment of advanced primary or metastatic breast cancer with an ineffective paclitaxel or anthracycline chemotherapy. With the widespread application of anthracyclines and taxanes in the treatment of breast cancer, an increasing number of patients develop resistance to anthracyclines and taxanes, which has become an urgent problem in clinical practice. As a new-generation oral fluorouracil drug, capecitabine selectively acts on tumor cells with a high expression of thymidine phosphorylase. Capecitabine has high effectiveness, low toxicity, and convenient administration. Li et al. conducted a phase II study on the combination of capecitabine and cisplatin in the treatment of mTNBC patients pretreated with anthracycline and taxane and found that the combination of capecitabine and cisplatin had significant activity in mTNBC patients, and side effects were acceptable [40].

## TNBC targeted therapy and potential treatment regimens

Due to the high heterogeneity of TNBC, it is particularly difficult to discover new therapeutic targets and perform targeted therapy. Currently, there are a large number of ongoing clinical trials targeting specific receptors or on targeted therapies of TNBC based on immunohistochemical staining results.

### Epidermal growth factor receptor (EGFR)

Nielsen et al. performed DNA microarray analysis on a large number of BLBC samples and found that approximately 60% of BLBC samples highly expressed EGFR [41]. The statistical results of Livasy et al. further confirmed that approximately 70–78% of basal-like TNBC samples highly expressed EGFR. Therefore, it is speculated that EGFR may be a therapeutic target in TNBC [42]. However, a randomized phase II trial (NCT00232505) selected 120 TNBC patients and found that cetuximab treatment alone had a response rates (RRs) less than 6%, and the combination of cetuximab and carboplatin had only 17% [43]. Therefore, although the preclinical study data strongly supported using EGFR as a potential target for TNBC targeted therapy, the clinical experimental data showed that the EGFR-targeted treatment for TNBC did not achieve the expected results. Using single-cell RNA sequencing (RNA-seq) data from a public resource, Cho et al. [44] recently identified an ERBB pathway-activated triple-negative cell population. The differential expression of three subtyping marker genes (ERBB2, ESR1, and PGR) was not changed in the bulk RNA-seq data, but the single-cell transcriptomes showed intratumor heterogeneity. This result indicates that ERBB signaling is activated through an indirect route and that the molecular subtype is changed at the single-cell level. The results of EGFR signaling pathway analysis in TNBC patients showed that the EGFR downstream signaling pathways were still activated in most patients after EGFR-targeted treatment, suggesting that there might be other pathways involved in a bypass activation. As a result, EGFR-targeted treatment alone cannot achieve significant efficacy. Based on the above results and the gene expression profiling analysis of Lehmann et al. [13], we speculate that the use of growth factor inhibitors in BL-2, M, and MSL subtypes combined with other downstream signal transduction inhibitors (PI3K, MAPK, and Scr inhibitors) might achieve better results.

### PARP inhibitors

PARP is a class of DNA repair enzymes. Its major function is to maintain genome stability, repair DNA, and participate in cell cycle progression and apoptosis [45]. PARP-1 is one of the most important enzymes in the PARP family and plays a vital role in DNA repair. Inhibition of PARP will lead to the loss of DNA repair function and thus induce apoptosis. PARP inhibitors can significantly enhance the therapeutic effects of radiotherapy and chemotherapy [46]. PARP inhibitors have significant antitumor effects on *BRCA1/2*-deficient tumors, and the inhibition effect on *BRCA1*-mutant tumors is 100–1000 times higher than in tumors without such mutations [47]. Up to 19.5% of TNBC patients carry *BRCA1/2* mutations, and black and Hispanic populations have a high likelihood of carrying *BRCA1/2* mutations [48, 49]. Therefore, PARP inhibitors are expected to be used in the targeted therapy of TNBC patients with *BRCA1* mutations. Unfortunately, recent clinical studies did not observe ideal treatment efficacy. The administration of olaparib, a PARP inhibitor, did not generate a significant difference in response rate between TNBC patients with and without *BRCA1/2* mutations [50]. Therefore, it was speculated that other DNA repair mechanisms might exist in TNBC patients that make these patients insensitive to PARP inhibitors alone. Studies have provided a preclinical rationale for the combined use of a DNA damaging agent with PI3K inhibitors by demonstrating that in addition to regulating cell growth, metabolism, and survival, PI3K also stabilizes double-strand breaks by interacting with the homologous recombination complex and, in effect, creates a BRCA-deficient state [51]. PI3K blockade promotes homologous recombination deficiency by downregulating BRCA1/2 and thus sensitizing BRCA-proficient tumors to PARP inhibition. To capitalize on these findings, a phase I study of the pan-PI3K inhibitor BKM120 (Novartis®) in combination with the PARP inhibitor olaparib in patients with metastatic TNBC is ongoing (NCT01623349). BKM120 would be expected to create a BRCA mutant-like tumor state, thus making the tumor susceptible to PARP inhibition [52]. According to the gene expression profiling analysis of Lehmann et al. [13], the top gene ontologies for the BL-1 subtype are heavily enriched in cell cycle and cell division components and pathways (cell cycle, DNA replication reactome, G2 cell cycle pathway, RNA polymerase, and G1 to S cell cycle), suggesting that PARP inhibitors and DNA synthetic inhibitors might be suitable for treatment.

### Androgen receptor (AR)

AR is expressed in both normal breast tissues and breast cancer tissues, but the levels are significantly different in different breast cancer tissues. AR expression is positive in approximately 10–15% of TNBC patients [53]. The LAR-subtype TNBC is defined as AR positive [13, 54]. Although there are relatively few studies on the roles of AR in breast cancer, Doane et al. compared 99 breast cancer patient samples and eight different breast cancer cell lines and discovered a cell line (MDA-MB-453) that shares traits with the LAR subtype. They carried out preclinical studies on MDA-MB-453 and found that it exhibited androgen-dependent growth. The proliferation of MDA-MB-453 can be inhibited by AR antagonism (flutamide). They therefore proposed a targeted therapy regimen for LAR-subtype TNBC patients by blocking AR [55]. Gucalp et al. performed antiandrogen therapy on LAR-subtype TNBC patients and found that this group of patients could benefit from antiandrogen treatment [56]. A phase II clinical trial using bicalutamide, a targeted AR inhibitor, for the treatment of breast cancer patients with positive AR and negative ER and PR expression showed a 19% clinical benefit rate (CBR) [56]. Traina et al. obtained a 25% CBR by using enzalutamide, an AR inhibitor, to treat AR-positive TNBC patients [57]. In addition to expression of the AR, the LAR-subtype cell lines have a high rate of PIK3CA activating mutations and exhibit strong sensitivity to PI3K inhibitors [13]. The coevolution of PIK3CA mutations with AR dependency is similar to ER-positive breast cancers, which have a high frequency of PIK3CA mutations [58, 59]. Preclinical data show that the combination of bicalutamide with a PI3K inhibitor produces an additive/synergistic effect, specifically in LAR cell lines. Therefore, this new targeted AR regimen is expected to be further developed, but more experimental support is needed, and the role of AR in the tumorigenesis of TNBC should be further explored.

### Estrogen receptor ER-ɑ36

TNBC cells, being negative for ER, PR, and HER2 expression, are generally believed to not have intracellular estrogen signal transduction. They are insensitive to endocrine therapy and lack known therapeutic targets. Wang et al. first discovered, cloned, and identified a new estrogen receptor, ER-α36, whose molecular weight is 36 kDa. This newly discovered ER is very different from the commonly studied ER-α66. Compared with ER-α66, ER-α36 lacks the transcriptional activator domains AF-1 and AF-2 but retains the DNA-binding domains and the domains of some dimeric ligands [60]. ER-α36 is mainly expressed in the cytoplasm and cell membrane, and its expression can be detected in both ER-positive and ER-negative breast cancer cells. Therefore, ER-α36 is a membrane-expressed ER that can rapidly mediate the transduction of estrogen and antiestrogen signaling in ER-positive and ER-negative breast cancer cells [61]. Zhang et al. studied the signaling mechanisms of ER-α36 in the TNBC cell lines MDA-MB-231 and MDA-MB-436 and found a positive feedback loop of EGFR and ER-α36 in TNBC, indicating that ER-α36 might be a potential target for the treatment of TNBC [62]. There is still a lack of support from clinical trials, and potential treatment programs remain to be explored.

### Immunotherapy

Tumor cells can evade recognition and destruction by the host immune system through the immune checkpoint system; thus, blocking the immune checkpoint system is a promising treatment strategy for achieving effective antitumor immunity. Programmed cell death-ligand 1 (PD-L1) is a 40-kDa transmembrane protein [63]. Under normal circumstances, the immune system reacts to foreign antigens that accumulate in the lymph nodes or spleen and promotes antigen-specific T cell proliferation. Programmed cell death protein 1 (PD-1) binds to PD-L1 and can transmit signals to inhibit T cell proliferation and promote T cell depletion. Through binding of PD-L1 to PD-1 on the surface of T cells, tumor cells transmit inhibition signals to T cells [64]. In one study, 59% of TNBC patients highly expressed PD-L1, 70% of patients had high PD-1 expression, and 45% of patients had high expression of both PD-L1 and PD-1. In addition, the expression of PD-L1 and PD-1 is associated with the degree of tumor lymphocyte infiltration and tumor histological grade [65, 66]. Similarly, Sun et al. conducted PD-L1 immunohistochemistry on 218 TNBC samples and found that TNBC cells expressed PD-L1, indicating that PD-L1 might be a potential TNBC immunotherapeutic target [67]. A 2016 clinical study on the treatment of TNBC using pembrolizumab, an anti-PD-1 monoclonal antibody, showed that the overall response rate (ORR) was 18.5% (95% CI, 6.3–38.1) in the 27 patients whose antitumor activity was evaluable. The responses were as follows: complete response, one case (3.7%); partial response, four cases (14.8%); stable disease, seven cases (25.9%); and progressive disease, 13 cases (48.1%) [68]. Similarly, a 2017 phase I clinical study using the anti-PD-L1 monoclonal antibody atezolizumab for treatment of TNBC showed that approximately 10% of TNBC patients experienced a lasting effect from treatment [69]. Although the CBR of immune checkpoint inhibitors targeting PD-L1/PD-1 was relatively low, some patients had a good prognosis and significantly increased OS rates. Therefore, the current major challenge is how to improve the response of TNBC patients to anti-PD-1/PD-L1 treatment and to convert nonresponders into responders. Such an improved treatment will help reduce the number of deaths and bring new hope to patients with advanced/metastatic TNBC [70]. In addition, there is an association between the immune response and the Ras/MAPK pathway in TNBC. One study has indicated that the Ras/MAPK pathway negatively regulates antitumor immunity by affecting antigen presentation, including that of MHC-I, MHC-II, and PD-1, and it was verified that a combination of MEK inhibition and PD-1/PD-L1 antibodies increased the effect of treatment in a murine syngeneic tumor model [71].

CTLA-4 inhibits T cell activation by binding to costimulatory molecules (such as CD80 and CD86) [72]. Ipilimumab, an anti-CTLA-4 antibody, has been approved by the US Food and Drug Administration (FDA) for the treatment of advanced melanoma. The ORR of melanoma patients who received ipilimumab monoclonal antibody treatment was 11% [73]. A phase I clinical study (NCT01927419) showed that, as the first-line treatment, the combination of ipilimumab and nivolumab (PD-1 antibody) for the treatment of advanced melanoma could increase the ORR to 61% [74]. Further study (NCT01927419) demonstrated that compared with monotherapy, the combination therapy significantly improved the ORR in patients with advanced melanoma, and the 2-year OS rate also significantly increased (63.8% for ipilimumab and nivolumab combination therapy vs. 53.6% for ipilimumab alone). However, the incidence of grade 3–4 adverse reactions in the combination therapy group was also significantly higher than that in the monoclonal antibody treatment group (59% vs. 20%). Grade 3–4 adverse events mainly included colitis and diarrhea [75]. Liu et al. used the combination of MUC1 mRNA nanovaccine and anti-CTLA-4 monoclonal antibody to treat TNBC and achieved a significant cell-killing effect in TNBC 4 T1 cells and observed an inhibitory effect on tumor growth in mice [76]. In a TNBC metastasis mouse model, Bernier et al. [77] significantly extended the survival time of mice using the combination of DZ-2384, a novel microtubule-targeting small-molecule compound, and CTLA-4 inhibitor. Therefore, optimizing the combination regimen may be the key to targeted CTLA-4 immunotherapy for TNBC.

Other immunotherapeutic methods include specific chimeric antigen receptor T cell (CAR-T) therapy. Song et al. found that CAR-engineered T cells targeting folate receptor (FR) α had highly efficient, specific killing and inhibition effects on FRα-expressing TNBC cells in vitro. In addition, they infused human CAR-T cells targeting FRα into immunodeficient mice carrying MDA-MB-231 tumor xenografts and found that the tumor growth was significantly inhibited [78]. Mesothelin is a membrane-bound glycoprotein. Its expression in normal human tissues is limited to mesothelial cells, and it is highly expressed in solid tumor tissues such as TNBC. Therefore, mesothelin might also be a new target for CAR-T treatment of TNBC [79]. AXL is a receptor tyrosine kinase that was first discovered in chronic myeloid leukemia patients together with two other kinases, Tyros and MER. AXL belongs to the TAM (Tyros, AXL, MER) family. Studies have shown that AXL is highly expressed on the cell surface of MDA-MB-231 in TNBC. AXL-CAR-T cells were constructed for in vitro cell-killing assays, and the results showed that AXL-CAR-T cells had a significant killing effect on MDA-MB-231 cells. AXL-CAR-T cells significantly inhibited the growth of subcutaneous xenografts of MDA-MB-231 cells [80].

## Summary and outlook

Compared with other breast cancer subtypes, TNBC is highly invasive and has a high early recurrence rate. Patients usually relapse within 5 years after surgery, with a very poor overall prognosis. Due to negative expression of ER, PR, and HER2, TNBC is insensitive to endocrine treatment and targeted therapies. Only very limited treatment regimens are available for TNBC, with generally poor efficacy. New therapies are urgently needed.

LAR-subtype TNBC has positive AR expression, but the mechanism and clinical significance of AR in TNBC are still controversial, and whether AR can be used as a prognostic indicator of TNBC remains to be further studied. It is worth noting that the mutation load of the LAR subtype is relatively high, mainly in *PIK3CA*, *CDH1*, *PTEN*, and *TP53* gene mutations in PI3K signaling genes. Therefore, targeting the PI3K signaling pathway may become a new therapeutic target for LAR-subtype TNBC. M-subtype TNBC samples have high expression of PDGFR, but this subtype is not sensitive to the corresponding targeted therapy. Whether M-subtype TNBC has other regulatory mechanisms causing drug resistance remains to be explored in-depth. The MSL subtype overexpresses angiogenesis-related receptors PDGFR and VEGFR, which might make them susceptible to antiangiogenic therapy. High expression of immune-related markers and of immune checkpoint inhibitor genes are the main differences between the IM subtype and other TNBC subtypes. Therefore, the IM subtype is likely to benefit from immune checkpoint inhibitor treatments.

All TNBC subtypes besides MSL show a high frequency of MYC gene amplification, and the BL1 and M subtypes also show corresponding mRNA overexpression. Selective inhibition of CDK1/2 and the core component BUD31 of restriction enzymes can induce apoptosis of TNBC tumor cells overexpressing MYC, suggesting that TNBC, especially the BL1 and M subtypes, might benefit from CDK1/2 and restriction enzyme inhibitor treatment. In addition, new targeted therapeutic measures can be developed based on mutations of different TNBC subtypes and types with abnormal gene copy numbers. For example, the BL1 subtype has high genomic instability, with *TP53*, *BRCA1/2*, and *RB1* gene deletions and *PPAR1* gene amplification, suggesting that the BL1 subtype may be sensitive to PARP inhibitors. The expression levels of RB1, CDK4, and CDK6 are related to the sensitivity of CDK4/6 inhibitors; LAR and MSL subtypes with low expression of CDK4 and CDK6 mRNA but high expression of RB1 may be sensitive to CDK4/6 inhibitors.

BLIA and BLIS are TNBC subtypes with opposite prognoses. BLIA has a better prognosis than LAR, MES, and BLIS, while BLIS has the worst prognosis. This difference suggests a possible correlation between the expression of immune signals in TNBC tumor cells and drug resistance and prognosis. In the BLIA type, signal transduction pathways associated with immune cells, such as the NK cell pathway, B cell receptor pathway, DC pathway, T cell receptor signal pathway, and the IL-12 and IL-7 pathways, are significantly enriched, while the expression levels of STAT, CTLA4, CXCL9, IDO1, CXCL11, RARRES1, GBP5, and CXCL10/13 are significantly increased. CXCL10 belongs to the druggable genome, so it is expected to become a pharmaceutical target. In addition, STAT inhibitors, cytokine or cytokine receptor antibodies, and ipilimumab (recently FDA-approved CTLA4 inhibitor) [81] might be used for the treatment of BLIA subtype TNBC. In the BLIS type, almost all immune cell signal transduction pathways are inhibited, while the expression levels of the *ELF5*, *HOHMAD1*, *FOXC1*, *VTCN1*, and *SOX6*, and *SOX10* genes are significantly increased. It was speculated that PD-1 or VTCN1 antibody could be used for targeted immune checkpoint treatment. The integrated analysis of DNA and mRNA expression data of TNBC patient samples from four different subtypes, LAR, MES, BLIA, and BLIS, showed that CDK1 was amplified in all four TNBC subtypes (BLIA subtype had the highest expression). Therefore, CDK1 may be a potential TNBC therapeutic target [19].

At the same time, with the refinement of TNBC subtypes, the new use of old drugs has become an important research direction to improve the efficacy of TNBC. Clinical trial results have shown that BL1 and BL2 TNBC patients have higher clinical remission rates for taxanes than MSL and AR subtypes; when these drugs are combined with anthracyclines, BL1 subtype patients with TNBC can achieve higher pCR rates; in addition, patients with BL1 subtype TNBC are more sensitive to platinum drugs. It is suggested that patients with BL1 TNBC combined with taxanes, anthracyclines, and platinum drugs may achieve a better clinical response rate. Of course, specific drug selection and drug delivery strategies require more clinical trial results to validate. We have classified different types of chemotherapeutic and antibody drugs and speculated that they may be suitable for TNBC subtypes (Table 3).

**Table 3:** Potential therapeutic strategies and targeted drugs used in particular subtypes of triple negative breast cancer

TNBC subtype	Therapeutic strategies	Therapeutic targeted drugs
BL1 (basal-like 1)	Inhibit cell proliferation and DNA damage response	**Mitosis inhibitors** (Paclitaxel, Docetaxel, Ixabepilone, Nab-Paclitaxel, Vinorelbine) **Cytostatics** (Cisplatin, Carboplatin, Nedaplatin, Eptaplatin, Oxaliplatin, Lobaplatin, Satraplatin, Mercaptopurine) **PARP inhibitors** (Olaparib, Rucaparib, Talazoparib, Niraparib) **DNA Synthetic inhibitors** (Topotecan, Irinotecan, Camptothecin, Doxorubicin, Daunorubicin, Mitomycin)
BL2 (basal-like 2)	Inhibit TP63, EGFR, and MET signaling	**Cytostatics** (Cisplatin, Carboplatin, Nedaplatin, Eptaplatin, Oxaliplatin, Lobaplatin, Satraplatin, Mercaptopurine) **PARP inhibitors** (Olaparib, Rucaparib, Talazoparib and Niraparib) **Growth Factor inhibitors** (Erlotinib, Gefitinib, Afatinib, Osimertinib, Olmutinib, Nazartinib, Avitinib, lapatinib, Cetuximab, Panitumumab, Vandetanib, Bevacizumab, Pertuzumab, Ramucirumab, Trastuzumab, Axitinib, Cabozantinib, Ceritinib, Crizotinib, Lenvatinib, Nilotinib, Pazopanib, Regorafenib, Sorafenib, Sunitinib) **mTOR inhibitors** (rapamycin, everolimus, RapaLink-1)
IM (immunomodulatory)	Inhibit immune signaling	**Cytostatics** (Cisplatin, Carboplatin, Nedaplatin, Eptaplatin, Oxaliplatin, Lobaplatin, Satraplatin, Mercaptopurine) **PARP inhibitors** (Olaparib, Rucaparib, Talazoparib and Niraparib) **Immune checkpoint inhibitors** (Ipilimumab, Nivolumab)
M (mesenchymal)	Inhibit EMT, Wnt, PI3K, mTOR, Scr, TGFβ, IGF1R, Notch	**Growth Factor inhibitors** (Erlotinib, Gefitinib, Afatinib, Osimertinib, Olmutinib, Nazartinib, Avitinib, lapatinib, Cetuximab, Panitumumab, Vandetanib, Bevacizumab, Pertuzumab, Ramucirumab, Trastuzumab, Axitinib, Cabozantinib, Ceritinib, Crizotinib, Lenvatinib, Nilotinib, Pazopanib, Regorafenib, Sorafenib, Sunitinib) **mTOR inhibitors** (Rapamycin, Everolimus, RapaLink-1) **Scr inhibitors** (Bosutinib, Dasatinib) **PI3K inhibitors** (Idelalisib)
MSL (mesenchymal stem-like)	Inhibit EMT, Wnt, TGFβ, MAPK, Rac, PI3K, mTOR, Scr, PDGF	**Growth Factor inhibitors** (Erlotinib, Gefitinib, Afatinib, Osimertinib, Olmutinib, Nazartinib, Avitinib, lapatinib, Cetuximab, Panitumumab, Vandetanib, Bevacizumab, Pertuzumab, Ramucirumab, Trastuzumab, Axitinib, Cabozantinib, Ceritinib, Crizotinib, Lenvatinib, Nilotinib, Pazopanib, Regorafenib, Sorafenib, Sunitinib) **mTOR inhibitors** (rapamycin, everolimus, RapaLink-1) **PI3K inhibitors** (Idelalisib) **MAPK inhibitors** (Trametinib, Dabrafenib) **Scr inhibitors** (Bosutinib, Dasatinib)
LAR (luminal androgen receptor)	Inhibit AR signaling, FOXA1, and ERBB4 signaling	**Nonsteroidal antiandrogens** (bicalutamide) **mTOR inhibitors** (rapamycin, everolimus, RapaLink-1) **PI3K inhibitors** (Idelalisib)

As a new tumor treatment regimen, immunotherapy has uncertain therapeutic effects on different TNBC subtypes, and more preclinical experimental data are needed. In TNBC immunotherapy targeting PD1/PDL-1, in addition to research and development of new targeted antibodies, studies have found that tumor-associated macrophages (TAMs) derived from peripheral-blood mononuclear cells were recruited into the TNBC microenvironment. By secreting inhibitory cytokines, the functional effects of tumor-infiltrating lymphocytes are attenuated, and regulatory T cells are increased to promote tumor growth and development. Interestingly, TAMs can simultaneously upregulate PD-1 and PD-L1 expression in the tumor environment. Therefore, targeting TAMs to improve the efficacy of PD1/PDL-1-targeting drugs might be a feasible new idea [82]. In addition, the modification of relevant CAR-T immune cell therapy targets and safety evaluation of such therapies need to be supported by more clinical data.

Undoubtedly, recent advances have been made in understanding TNBC as a disease with intrinsic molecular subtypes and immunological heterogeneity, recognizing the variety of clinical phenotypes. This new scenario demands an urgent comprehensive subclassification that incorporates immune-molecular signatures for more targeted and effective treatment. Although targeted inhibitors and checkpoint inhibitors have recently been incorporated in some settings, cytotoxic chemotherapy remains the mainstay therapy against TNBC, resulting in different outcomes for patients with similar clinicopathologic features.

A more complete accessible panel of immunohistochemical molecular subtypes has improved decisions in the treatment of TNBC. Additionally, in many cases, more precise molecular classification of tumors has been proposed to predict survival and response to chemotherapy, allowing for personalized approaches, such as the need for dose escalation and incorporation of new antitumor agents into the standard regimen, and for new treatment options, such as CAR-T immune cell therapy, checkpoint inhibitors, and molecular targeted inhibitors.

Formerly considered a disease unapproachable with molecular therapy, TNBC has recently been the center of successful investigations for incorporation of new targeted therapies due to intrinsic molecular TNBC subtyping and accurate classification and prediction of prognosis improvements. Considering the proposed subtypes and their molecular variations as defined by specific biomarkers and the current chemotherapy, immunotherapy, and targeted inhibitor combination options, great advances have been achieved in TNBC treatment.

## Data Availability

Not applicable
